# Expression and clinical significance of long non-coding RNA *HNF1A-AS1* in human gastric cancer

**DOI:** 10.1186/s12957-015-0706-3

**Published:** 2015-10-15

**Authors:** Yuan Dang, Fenghua Lan, Xiaojuan Ouyang, Kai Wang, Youdong Lin, Yinghao Yu, Lie Wang, Yu Wang, Qiaojia Huang

**Affiliations:** Department of Experimental Medicine, Fuzhou General Hospital (Dongfang Hospital), 156 North Xi-er Huan Road, Fuzhou City, Fujian Province 350025 China; Department of General Surgery, Fuzhou General Hospital (Dongfang Hospital), 156 North Xi-er Huan Road, Fuzhou City, Fujian Province 350025 China; Department of Pathology, Fuzhou General Hospital (Dongfang Hospital), 156 North Xi-er Huan Road, Fuzhou City, Fujian Province 350025 China

**Keywords:** Gastric cancer, lncRNA, *HNF1A-AS1*, Biomarker

## Abstract

**Background:**

Increasing evidence has demonstrated that long non-coding RNAs (lncRNAs) play essential roles in the occurrence and development of human cancers, including gastric cancer (GC). However, the functional and clinical significance of lncRNAs are still poorly understood.

**Methods:**

In this study, the expression of LncRNA HNF1A antisense RNA 1 (*HNF1A-AS1*) was first examined by lncRNAs microarray analysis in 6 GC tissues, and was then further verified by real-time quantitative reverse transcription PCR (qRT-PCR) both in 3 GC cell lines and 161 cases of GC tissues. We also evaluated the association between *HNF1A-AS1* expression and clinicopathological features of patients with GC.

**Results:**

LncRNAs microarray analysis results exhibited that *HNF1A-AS1* was downregulated in GCs tissues (mean fold change 2.06, *p* < 0.05), which was further confirmed by qRT-PCR. The results from qRT-PCR showed that the expression of *HNF1A-AS1* was not only downregulated in three GC cell lines (AGS, BGC-823, and MKN-45) relative to that in a normal gastric mucosal epithelial cell line (GES-1), but also decreased in GC tissues relative to that in paired adjacent non-neoplastic tissues (low expression, 94 of 161; low expression rate, 58.38 %). Furthermore, low *HNF1A-AS1* expression was associated with tumor size/diameter (*p* = 0.005, multivariate analysis), levels of serum carcinoembryonic antigen (CEA), and carbohydrate antigen 19-9 (CA19-9), and RRM1 expression in tissue samples (*p* = 0.028, *p* = 0.009, and *p* = 0.006, respectively).

**Conclusions:**

Taken together, our data indicate that lncRNA *HNF1A-AS1* may be a regulator of GC, and thus, it may have potential as a novel biomarker and treatment target for this type of cancer.

## Background

Gastric cancer (GC) is one of the most common cancers and can occur in any section of the human stomach. These tumors are frequently malignant and have the ability to invade perineural tissue [1]. Data from the latest global annual survey have shown that there are almost 1 million new cases and 740,000 deaths from this cancer per year worldwide. The incidence of GC in China is higher than that in most countries in the world, with more than 400,000 new cases each year and approximately 300,000 deaths. Studies have shown that if this illness can be diagnosed at an early stage, good prognosis can be achieved through complete surgical resection of the primary tumor tissue [2]. Unfortunately, patients with GC show few symptoms in the early stages of disease and diagnosis is usually difficult. The 5-year survival rate of GC at advanced stages remains poor and is stubborn at approximately 30 % [3]. Cancer biomarkers allow cancer to be diagnosed and can serve as therapeutic targets, as well as can be used to explore the pathogenesis of disease; therefore, identification of cancer biomarkers would be of great value.

Long non-coding RNAs (lncRNAs) are non-coding RNAs greater than 200 nucleotides [4]. These RNAs do not encode proteins and are the main components of the human transcriptome [5]. Emerging evidence indicates that lncRNAs are important regulatory molecules whose main functions are related to modulation of gene expression networks [6]. Many of these networks are closely associated with cancer development and progression [6–9]. Data from different groups have determined that changes in expression of lncRNAs are related to the pathogenesis of diverse human cancers. Recent findings from Wang et al., Song et al., and Shao et al. have demonstrated that changes in the expression profiles of lncRNAs are linked to gastric cancer carcinogenesis and progression and that lncRNAs could be used as early biomarkers or as treatment targets for GC [10–12].

In order to identify the abnormal expression of tumor-related LncRNAs and those LncRNAs with potential values as biomarkers, in this study, the lncRNAs expression profile microarray analysis was performed in six GC tissue and their paired adjacent non-neoplastic gastric tissues. LncRNA HNF1A antisense RNA 1 (*HNF1A-AS1*) was one of the LncRNAs that were identified to be downregulated in GC tissues by the microarray analysis. *HNF1A-AS1*, a single-exon gene at band 12q24.31 of chromosome 12, is a bidirectional lncRNA of 2455 nucleotides [13]. The transcriptional start site of this lncRNA is very close to that of hepatic nuclear factor 1 alpha gene (*HNF1A*; *HNF1A-AS1*’s start site is approximately 5 kb downstream of *HNF1A*) [13]. To our knowledge, the expression of *HNF1A-AS1* in GC tissues remains unreported. Therefore, in this work, we determined the expression levels of *HNF1A-AS1* in 3 GC cell lines and in 161 cases of GC tissues and their paired adjacent non-neoplastic gastric tissues. We also investigated the potential association between the expression of *HNF1A-AS1* and clinicopathological features of patients with GC. Our data illustrate the potential of this lncRNA as a novel biomarker and a treatment target for GC.

## Methods

### Tissue samples and clinical data collection

One hundred and sixty-one fresh paired tissue samples (GC tissue and non-neoplastic tissues collected 5 cm from the cancer’s edge) were obtained from Fuzhou General Hospital, Fujian, China, in 2014 and 2015. All of the samples were obtained immediately after surgical operation and preserved in RNAlater (Qiagen, Germany) at −80 °C until use. Serum samples used for detection of the serum tumor biomarkers and paraffin-embedded tissue samples used for detection of the immunohistochemical markers were also collected from the same patients. Every tissue sample was histopathologically diagnosed and confirmed by at least two pathologists. The standards for tumor-node-metastasis (TNM) stage and histological grade were in accordance with the guidelines of the International Union Against Cancer (UICC; 5th Ed) and the National Comprehensive Cancer Network’s (NCCN) Clinical Practice Guidelines in Oncology (V.1.2011), respectively. None of the patients received treatment prior to resection.

### Ethics statement

Informed consent was obtained from all individual participants included in the study, and this study was approved by the Ethics Committee of Fuzhou General Hospital.

### LncRNAs microarray assay

The lncRNAs microarrays used in this study were the Human LncRNAs Microarray V3.0 (Arraystar Inc., MD, USA), which was composed of lncRNAs and mRNAs from the human genome. lncRNAs microarray assay and the data analysis were performed by KANGCHEN Bio-tech (Shanghai, China) based on the instructions of the manufacturer.

### Cell culture

AGS, BGC-823, and MKN-45 GC cell lines and normal human gastric epithelial cell line (GES-1) were obtained from the American Type Culture Collection, Manassas, VA, Shanghai Institute of Biochemistry and Cell Biology, the Chinese Academy of Sciences in Shanghai, China, Japanese Cancer Research Bank and Beijing Cancer Institute (Beijing, China), respectively. The cells were grown in F12, RP1640, or DMEM medium (Invitrogen, Grand Island, NY, USA), all of which contained 10 % fetal bovine serum, in an incubator at 37 °C with 5 % CO_2._

### Total RNA extraction and qRT-PCR

Total RNAs were isolated from both tissues and cultured cells by using TRIzol reagent (Invitrogen). Reverse transcription was then performed by using a Reverse Transcription kit (Promega) in accordance with the instructions of the manufacturer. Real-time quantitative reverse transcription PCR (qRT-PCR) was carried out by using the SYBR Green Mix kit (Promega, Madison, WI, USA) in a Step One Real-time PCR System (ABI, Grand Island, NY, USA). The primers for 18s and *HNF1A-AS1* qPCR were as follows: (forward) 5′-TCAAGAAATGGTGGCTAT-3′ and (reverse) 5′-GCTCTGAGACTGGCTGAA-3′ for *HNF1A-AS1*; (forward) 5′-AGAAACGGCTACCACATCCA-3′ and (reverse) 5′-CACCAGACTTGCCCTCCA-3′ for 18s. The conditions for PCR were as follows: stage 1, 95 °C for 10 min; stage 2, 40 cycles at 95 °C for 15 s, 57 °C for 1 min; stage 3 (dissociation stage), 95 °C for 15 s, 60 °C for 15 s, 95 °C for 15 seconds. The recorded cycle threshold (Ct) values included both *HNF1A-AS1* and 18 s; the latter served as a control. The expression level of *HNF1A-AS1* was obtained by using the ΔCt method. Higher ΔCt values indicated lower *HNF1A-AS1* expression. The results were obtained from three independent experiments and expressed as the mean ± standard deviation (SD).

### Measurement of AFP, CA-125, CEA, and CA19-9 concentrations in the serum of patients with GC

The concentrations of alpha-fetoprotein (AFP), carcinoembryonic antigen (CEA), cancer antigen 125 (CA-125), and carbohydrate antigen 19-9 (CA19-9) were detected in the serum of all of 161 cases of this group patients by using the Quantitative Kit for Tumor Marker (Protein Chip-Chemiluminescence) (HealthDigit, Huzhou, China) with the HD-2001A ChipReader System (HealthDigit). In this study, the normal reference values for healthy individuals were <20.0 ng/ml, <5.0 ng/ml, <35.0 U/ml and <35.0 U/ml for AFP, CEA, CA-125, and CA19-9, respectively.

### IHC

We used immunohistochemistry (IHC) in paraffin-embedded sections to determine the expression of vascular endothelial growth factor (VEGF), human epidermal growth factor receptor 2 (C-erbB-2 or HER2), thymidylate synthase (TS), breast cancer 1 (BRCA1), excision repair cross-complementation group 1 (ERCC1), Ki67 antigen (Ki67), and ribonucleotide reductase subunit M1 (RRM1), synaptophysin (Syn), neuronal cell adhesion molecules (CD56), and chromogranin A (CgA); 150 cases of the paraffin-embedded tissue samples of this group patients were available for the IHC assay. IHC was performed by pathologists in accordance with the instructions of the manufacturers. The antibodies and other reagents for IHC used in this work were purchased from Maixin (Fujian, China) and ZSGB-BIO (Beijing, China). With the exception of Ki67, the intensity of staining was graded from 0 to 4, with 0 being no staining; 1, less than 25 % of cells positive; 2, 25 to 50 % of cells positive; 3, 51 to 75 % of cells positive; and 4, >76 % cells positive. For Ki67, the grades were directly judged as the percent positive for Ki67 expression. The slides were graded by at least two pathologists.

### Statistical analysis

Statistical Program for Social Sciences (SPSS) 16.0 software (SPSS, Chicago, IL, USA) and GraphPad Prism 5.0 (GraphPad Software, LaJolla, CA, USA) were used for all statistical analyses. One-way analysis of variance (ANOVA), the rank-sum test, and the Student’s *t* test were main statistical methods used in this work, the *p* < 0.05 was regarded as statistically significant.

## Results

### LncRNAs microarray analysis exhibits that *HNF1A-AS1* expression is downregulated in GCs tissues

**Fig. 1:**
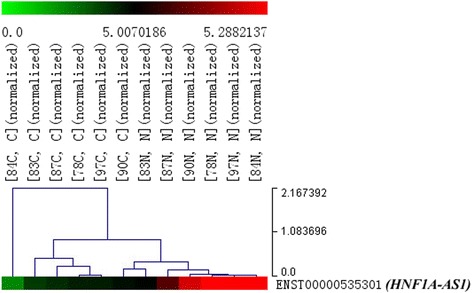
*HNF1A-AS1* was downexpressed in six GC tissues identified by lncRNAs microarray analysis. 84C, 83C, 87C, 78C, 97C, and 90C indicated six GC tissues (84, 83, 87, 78, 97, and 90 were the six cases of tissue number, *C* cancer); 83N, 87N, 90N, 78N, 97N, and 84N indicated the six corresponding paired adjacent non-neoplastic gastric tissues (*N* non-neoplastic gastric tissues). Cluster analysis based on the microarray results exhibited that *HNF1A-AS1* expression in GC tissues was downregulated compared with that of paired non-neoplastic gastric tissues (mean fold change 2.06, *p* < 0.05)

As shown in Fig. 1, the results of lncRNAs expression profile microarray analysis gotten from six GC tissues and their paired adjacent non-neoplastic gastric tissues exhibited that *HNF1A-AS1* expression was downregulated in all of the six GC tissues (mean fold change 2.06, *p* < 0.05), which indicated that this lncRNA may have the potential to be one of the tumor-related LncRNAs. Thus, we chose it for further analysis.

### qRT-PCR verification further confirms that *HNF1A-AS1* expression is downregulated in GC cell lines and tissues

**Fig. 2:**
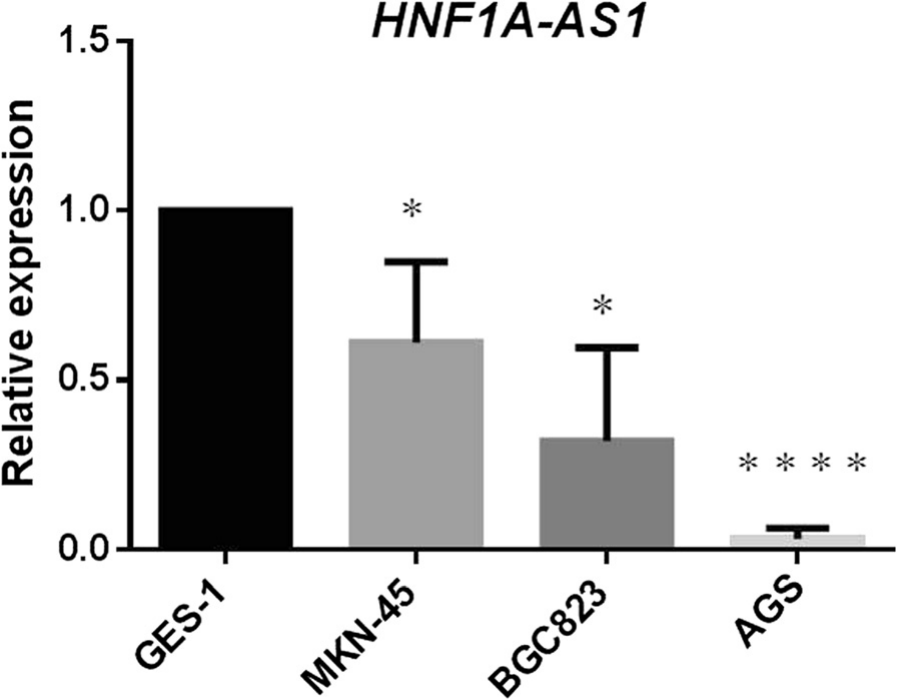
Decreased expression of *HNF1A-AS1* in GC cells relative to that in normal gastric epithelial cells. qRT-PCR was used to compare the expression of *HNF1A-AS1* in BGC-823, MKN-45, and AGS GC cells to that of non-neoplastic GES-1 cells. Data shown are from three independent experiments and expressed as the mean ± SD. *Asterisks* indicate values with a statistically significant difference versus the value in GES-1 cells. *p < 0.05, ****p < 0.0001. *GC* gastric cancer, *qRT-PCR* quantitative real-time PCR, *SD* standard deviation

**Fig. 3:**
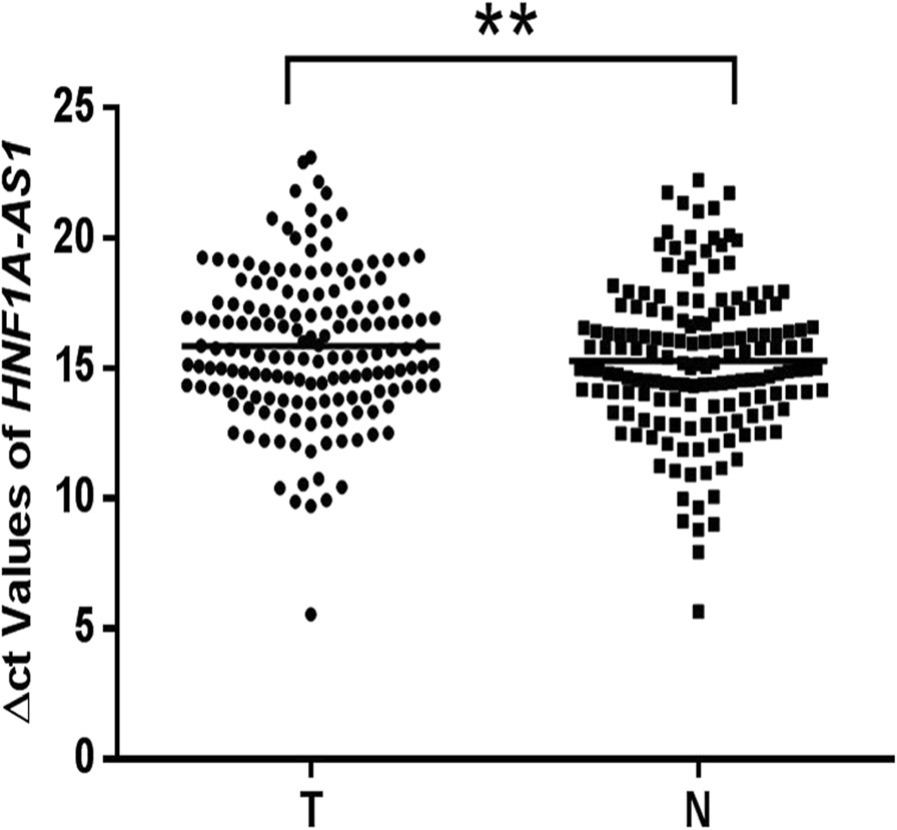
Expression of *HNF1A-AS1* in neoplastic and non-neoplastic gastric tissues. The expression of *HNF1A-AS1* in GC tissues was compared to that in adjacent non-neoplastic tissues (*n* = 161, **p < 0.01). *T* GC tissues, *N* non-neoplastic tissues. Data shown are from three independent experiments and expressed as the mean ± SD (∆Ct value). *GC* gastric cancer, *SD* standard deviation

We first investigated the expression of lncRNA *HNF1A-AS1* in three GC cell lines: AGS, BGC-823, and MKN-45. By using qRT-PCR, we confirmed that the expression of *HNF1A-AS1* was significantly decreased in all three GC cell lines relative to that in normal gastric epithelial cells (GES-1 cells; AGS, *p* < 0.0001; BGC-823, *p* < 0.05; MKN-45, *p* < 0.05; Fig. 2). We also examined *HNF1A-AS1* expression in GC tissue samples and paired adjacent non-neoplastic tissue samples. Our results showed that *HNF1A-AS1* was downregulated in 58.38 % (94/161) of GC tissues relative to that in the non-neoplastic tissues (*p* < 0.01, Fig. 3). Taken together, these data indicate that *HNF1A-AS1* is downregulated in human GC.

### Association of *HNF1A-AS1* expression with clinicopathological features of patients with GC

To assess the clinical significance of *HNF1A-AS1*, we analyzed the relationship between its expression and clinicopathological features of patients with GC. As shown in Tables 1 and 2, the univariate analysis exhibited that the expression of *HNF1A-AS1* was associated with tumor size/diameter (*p* = 0.002), invasion depth (T stages, *p* = 0.032), lymphatic metastasis (N stages, *p* = 0.006), venous invasion (*p* = 0.001), and perineural invasion (PNI, *p* = 0.014). However, after controlling affect factors including age, sex, tumor location, tumor size/diameter, differentiation, invasion (T stages), lymphatic metastasis (N stages), venous invasion, and PNI, the multivariate analysis further confirmed that tumor size persisted to be significantly correlated to HNF1A-AS1 expression (*p* = 0.005), which meant that patients with tumor size ≥5 cm had lower levels of HNF1A-AS1. These data suggest that *HNF1A-AS1* may play roles during cancer occurrence and progression and may be a new biomarker of gastric cancer.

**Table 1:** Association of HNF1A-AS1 expression (ΔCt) with clinicopathological features of patients

Characteristics	No. of case (%)	Mean ± SD	*p* value
Age (years)			0.119
≥60	82(50.9)	16.17 ± 2.90	
<60	79(49.1)	15.47 ± 2.71	
Gender			0.688
Male	123(76.4)	15.78 ± 2.88	
Female	38(23.6)	15.99 ± 2.66	
Tumor location			0.560
Sinuses ventriculi	57(35.4)	16.06 ± 3.13	
Cardia	33(20.5)	15.87 ± 3.04	
Corpora ventriculi	45(28.0)	15.92 ± 2.50	
Others	26(16.1)	15.11 ± 2.36	
Diameter(cm)			*0.002*
≥5	73(45.3)	16.59 ± 2.8	
<5	88(54.7)	15.2 ± 2.70	
Differentiation			0.396
Well	2(1.2)	16.15 ± 0.71	
Moderate	34(21.1)	15.24 ± 2.66	
Poor	125(77.7)	15.98 ± 2.87	
Invasion			*0.032*
T1	13(8.1)	16.13 ± 1.95	
T2	14(8.7)	14.09 ± 2.24	
T3	16(9.9)	14.85 ± 3.71	
T4	118(73.3)	16.13 ± 2.76	
Lymphatic metastasis			*0.006*
N0	38(23.6)	14.74 ± 2.77	
N1-3	123(76.4)	16.16 ± 2.76	
Venous invasion			*0.001*
Absent	86(53.4)	15.14 ± 2.58	
Present	75(46.6)	16.61 ± 2.90	
Perineural invasion (PNI)			*0.014*
Absent	89(55.3)	15.34 ± 2.77	
Present	72(44.7)	16.43 ± 2.79	
CEA			*0.028*
Normal	130(80.7)	15.59 ± 2.74	
High	31(19.3)	16.83 ± 2.97	
CA19-9			*0.009*
Normal	140(87.0)	15.60 ± 2.70	
High	21(13.0)	17.32 ± 3.22	
AFP			0.936
Normal	150(93.2)	15.82 ± 2.80	
High	11(6.8)	15.89 ± 3.25	
CA-125			0.494
Normal	154(95.7)	15.79 ± 2.84	
High	7(4.3)	16.55 ± 2.33	

Italicized values are significant at *p* < 0.05

**Table 2:** Comparison of the *p* values obtained from univariate and multivariate analyses of the association of HNF1A-AS1 expression (ΔCt) with clinicopathological features of patients

Characteristics	Univariate	Multivariate
	*p* value	*p* value
Age (years)	0.119	0.197
Gender	0.688	0.174
Diameter (cm)	0.002	*0.005*
Differentiation	0.396	0.603
Invasion	0.032	0.580
Lymphatic metastasis	0.006	0.235
Venous invasion	0.001	0.057
Perineural invasion (PNI)	0.014	0.192
Tumor location	0.560	0.342

Italicized values are significant at *p* < 0.05

### Association of *HNF1A-AS1* expression with serum or immunohistochemical markers of GC

*HNF1A-AS1* expression in GC tissues was associated with two serum markers of digestive tract tumors, CEA (*p* = 0.028) and CA19-9 (*p* = 0.009), but there was no significant association with other digestive tract cancer serum marker AFP or with the epithelial tumor marker CA125 (Table 1). The expression of *HNF1A-AS1* in tumor samples was also significantly correlated with one immunohistochemical markers of cancer, RRM1 (*p* = 0.006), but not with VEGF, C-erbB-2, TS, BRCA1, ERCC1, Ki67, CD56, Syn, or CgA (Table 3).

**Table 3:** Association of HNF1A-AS1 expression (ΔCt) with immunohisochemical features of GC tissue

Characteristics	No. of case (%)	Mean ± SD	*p* value
VEGF			0.809
0	15 (10.0)	15.27 ± 3.69	
1	22 (14.7)	15.57 ± 2.92	
2	33 (22.0)	15.83 ± 2.72	
3	59 (39.3)	15.94 ± 2.54	
4	21 (14.0)	16.39 ± 3.32	
C-erbB-2			0.312
0	129 (86.0)	15.70 ± 2.84	
1	5 (3.3)	16.37 ± 2.96	
2	4 (2.7)	15.77 ± 1.01	
3	12 (8.0)	17.30 ± 3.21	
TS			0.543
0	22 (14.7)	16.41 ± 2.45	
1	53 (35.3)	15.99 ± 2.97	
2	52 (34.7)	15.33 ± 2.73	
3	21 (14.0)	16.25 ± 3.34	
4	2 (1.3)	15.92 ± 1.82	
BRCA1			0.893
0	10 (6.7)	16.59 ± 1.83	
1	31 (20.7)	16.07 ± 2.91	
2	73 (48.6)	15.78 ± 2.57	
3	34 (22.7)	15.61 ± 3.57	
4	2 (1.3)	15.77 ± 5.01	
ERCC1			0.585
0	19 (12.7)	15.81 ± 2.39	
1	22 (14.7)	16.51 ± 3.61	
2	44 (29.3)	15.96 ± 2.85	
3	41 (27.3)	15.85 ± 3.05	
4	24 (16.0)	15.11 ± 2.02	
RRM1			*0.006*
0	81 (54.0)	15.79 ± 2.41	
1	10 (6.7)	13.27 ± 4.11	
2	17 (11.3)	15.51 ± 3.08	
3	35 (23.3)	16.98 ± 2.93	
4	7 (4.7)	15.48 ± 2.67	
Ki67			0.328
1	14 (9.3)	16.54 ± 1.67	
2	35 (23.3)	16.44 ± 2.79	
3	57 (38.0)	15.48 ± 2.97	
4	44 (29.4)	15.66 ± 3.02	
Syn			0.463
0	128 (85.3)	15.99 ± 2.95	
1	16 (10.7)	15.15 ± 2.17	
2	3 (2.0)	13.98 ± 0.87	
3	3 (2.0)	15.59 ± 3.06	
CD56			0.364
0	137 (91.4)	15.94 ± 2.86	
1	9 (6.0)	15.66 ± 3.09	
2	2 (1.3)	12.72 ± 0.84	
3	2 (1.3)	14.22 ± 0.97	
CgA			0.980
0	141 (94.0)	15.87 ± 2.91	
1	7 (4.7)	15.69 ± 1.83	
2	2 (1.3)	15.63 ± 3.26	

Italicized values are significant at *p* < 0.05

## Discussion

Several lines of evidence have demonstrated that changes in expression of lncRNAs play important roles in carcinogenesis and cancer development [14, 15] and that altered expression of lncRNA is known to be significantly associated with several clinicopathological features of human tumors, such as lymph node metastasis, TNM stage, distant metastasis, and the grade of tissue differentiation. For example, decreased expression of gastric cancer-associated transcript 1 (*GACAT1*) is significantly associated with metastasis, invasion, and tissue differentiation in patients with GC [16]. In addition, elevated expression of homeobox (HOX) transcript antisense RNA (*HOTAIR*), a long intergenic non-coding RNA, is associated with the grade of endometrial carcinoma (EC), metastasis, depth of myometrial invasion, and poorer overall survival [17]. Finally, low expression of *HMlincRNA717* in GC is associated with distal metastasis and invasion of the venous and nervous systems [12]. All of these findings are evidence of the important roles of lncRNAs in carcinogenesis and of the potential value in their clinical application.

In this study, we determined that the expression level of *HNF1A-AS1* in three gastric cancer cell lines was significantly decreased relative to that in normal gastric epithelial cells. More importantly, it was downregulated in 58.38 % of gastric cancer tissue samples relative to that in adjacent non-neoplastic tissues. The findings from Yang et al. show that this lncRNA was upregulated in esophageal adenocarcinoma (EAC) [13]. Carcinogenesis and cancer progression is a complex pathological process that is mediated by gene expression networks. Cancer occurrence in different organs or tissues may be regulated by differentially expressed levels of the same genes or the same lncRNAs [18, 19]. This may explain why *HNF1A-AS1* exhibits divergent expression profiles in different types of cancer cells. Further investigation may be necessary to reveal the precise molecular mechanism for these differences.

It is well known that cancer invasion and metastasis frequently occurs via three common pathways: lymph node metastasis, travel through the vasculature, and local seeding and growth. PNI is an additional means of invasion that is usually correlated with local recurrence. Because local recurrence is closely associated with poor prognosis, identifying patients at high risk of PNI has great clinical significance.

The prognosis and survival of patients with GC may also be affected by the size of the tumor, which is related to its time of growth. Smaller tumors exhibit less infringement into the stomach than do larger tumors. Moreover, increased depth of invasion by larger tumors increases the likelihood that the lymphatic system will be breached. Increasing lymph node metastasis allows the cancer to grow more easily and results in poor prognosis.

Both CEA and CA19-9 are well-known tumor markers that are usually upregulated in serum or tissue samples from patients with early or primary malignant tumors of the digestive tract. RRM1 is one of the three known human ribonucleotide reductase with the functions of regulating the cancer cell proliferation via mediating DNA synthesis and repair, and detection of the expression of RRM1 in GC tissues by IHC has been identified to have prognostic significance in patients with GC [20].

## Conclusions

In summary, we analyzed the association of the expression level of *HNF1A-AS1* with the clinicopathological features of patients with GC. Our results showed that the expression of *HNF1A-AS1* was significantly downregulated in both GC tissues and cell lines. Moreover, decreased expression was associated with tumor size/diameter and levels of serum CEA, CA19-9, and tissue RRM1. These findings indicate that *HNF1A-AS1* may be involved in suppression of gastric cancer occurrence and development and that it has the potential to serve as a novel treatment target and biomarker for the evaluation of disease severity in patients with GC. Because the sample size of this study was relatively small, further verification may be necessary to confirm *HNF1A-AS1*’s true clinical value.
